# The relationship between camouflaging and mental health: Are there differences among subgroups in autistic adults?

**DOI:** 10.1177/13623613231185402

**Published:** 2023-07-27

**Authors:** Wikke J van der Putten, Audrey JJ Mol, Tulsi A Radhoe, Carolien Torenvliet, Joost A Agelink van Rentergem, Annabeth P Groenman, Hilde M Geurts

**Affiliations:** 1 Leo Kannerhuis (Youz/Parnassia Group), The Netherlands; 2 University of Amsterdam, The Netherlands

**Keywords:** camouflaging, heterogeneity, mental health, psychiatric problems, subgroups

## Abstract

Camouflaging is hypothesized to play an important role in developing mental health difficulties. But this might not be true for everyone. It remains unclear for whom camouflaging is associated with mental health. In this preregistered study (AsPredicted #45095), we investigated whether (1) camouflaging and mental health were associated and (2) we could detect subgroups with a different association between camouflaging and mental health. For this study, 352 autistic adults aged 30–84 years filled in, among others, the Dutch Camouflaging Autistic Traits Questionnaire to measure camouflaging and the Symptom Checklist-90 Revised to measure mental health difficulties. We found a moderate correlation between camouflaging and mental health difficulties (*r* = 0.45). However, there was only a strong association between camouflaging and mental health in a small subgroup, while the association was small in most autistic adults. For varying levels of negative affect and (to a lesser extent) autism traits, the association between camouflaging and mental health differed, but not for biological sex, age, or educational level. Thus, while one should be careful with group-based conclusions regarding the (negative) impact of camouflaging, camouflaging can be important to consider in clinical practice, especially for people with a substantial level of negative affect.

**Lay abstract:**

When autistic people use strategies to hide their autistic characteristics, we call this camouflaging. Autistic adults suggested that camouflaging can result in mental health difficulties. That is, people who report to camouflage also report mental health difficulties. However, since there are many differences between autistic people, this relationship may also differ between subgroups. Therefore, in this study we investigated whether camouflaging and mental health difficulties are related and whether this relationship is equal for all autistic adults. For this study, 352 autistic adults aged 30–84 years filled in the Dutch Camouflaging Autistic Traits Questionnaire to measure camouflaging and the Symptom Checklist-90 Revised to measure mental health difficulties. We found that camouflaging was moderately related to mental health difficulties. This means that people who report more camouflaging also report more mental health difficulties. When we looked closer, we found that this relationship was strong for only a small subgroup of autistic adults. In most other autistic adults, there was a small or no relationship between camouflaging and mental health difficulties. Therefore, it is important that clinicians are aware of camouflaging and its possible relationship with mental health difficulties, but that they do not generalize the negative consequences to everyone.

## Introduction

We all sometimes hide sides of ourselves that we do not want everyone to see. When someone asks how you are doing, you might smile and say that you are fine while you are actually feeling down. In situations where we meet new people, we may pretend to like the same things or share similar opinions to make a connection. However, when you differ from the norm or when you are stigmatized, you may feel that you have to adjust or hide certain behaviors even more to not stand out (Han et al., 2022). In autism^1^ research, the term “camouflaging^2^” is often used when describing conscious or unconscious behaviors to hide or minimize the visibility of one’s autism traits (Hull et al., 2017; Libsack et al., 2021). Camouflaging includes strategies through which someone actively compensates for difficulties in social situations, portraying a non-autistic persona and trying to fit in with others in social situations (Hull et al., 2019). While some people may be able to put on a proverbial mask seemingly effortlessly, autistic adults often indicate that camouflaging requires a lot of energy and is a potential reason for developing mental health difficulties.

Some autistic adults report that camouflaging has positive consequences, such as that it can help with maintaining friendships, getting employment, and improving independence (Libsack et al., 2021; Livingston et al., 2019). For others, negative consequences of camouflaging are more present, that is, experiencing mental health difficulties such as general and social anxiety, suicidal thoughts, or depression (Cook et al., 2021). While we talk about consequences here, it is important to note that these studies are all cross-sectional and therefore we cannot draw conclusions about causality. Mental health conditions are more common in autistic adults than in non-autistic adults (Lai et al., 2019). Understanding how camouflaging plays a role in the development of mental health difficulties could improve the care for autistic adults. However, studies investigating the association between camouflaging and mental health difficulties vary widely (Cook et al., 2021). The results vary between different types of mental health difficulties that are investigated and how camouflaging is operationalized. Also, mental health is often measured with self-reported questionnaires, while using clinical interviews may provide different results. Finally, the heterogeneity among autistic adults can be another reason for the varying results. If we could identify for whom camouflaging is an important factor in the development of mental health problems, this could help in providing more adequate care. We discuss the following factors that we will investigate in the subsequent paragraphs: autism traits, biological sex, age, cognitive abilities, and emotional affect.

As camouflaging research originates from and resonates with the autism community, being autistic in itself or someone’s level of autistic traits are potentially important factors in camouflaging (Lai et al., 2021). Overall, studies suggest that having an autism diagnosis or having more autistic traits is associated with camouflaging (Hull et al., 2019; Livingston et al., 2019). That is, when someone has more autism traits, this person may use more strategies to hide these behaviors. In addition, autistic adults report that camouflaging is very exhausting as it requires a lot of energy to figure out how one should behave in different situations (Bargiela et al., 2016; Hull et al., 2017; Livingston et al., 2019). For some autistic adults, camouflaging challenges their self-perception (Hull et al., 2017). When they act in a non-authentic way and pretend not to be autistic, they feel like they are losing their own identity. Therefore, the level of autism traits could be important for whether camouflaging is associated with mental health difficulties.

Biological sex is another important aspect when investigating camouflaging. That is, autistic women often report camouflaging as a reason for late diagnoses and for developing psychiatric problems (Bargiela et al., 2016). Most studies indeed showed that autistic women report more camouflaging compared with autistic men (for a more elaborate discussion see Cook et al., 2021). However, findings are mixed about whether the association between camouflaging and mental health differs between men and women. Thus, we included biological sex as a factor to gain more clarity about whether there is a difference in how camouflaging and mental health are related across sexes.

In addition, age is a relevant factor when investigating mental health difficulties, as older autistic adults meet the criteria of fewer psychiatric disorders compared with younger autistic adults (Lever & Geurts, 2016). The authors hypothesized that older adults, themselves as well as their environment, may have accepted their (social) differences, which could explain the decline in psychiatric problems at an older age. A decrease in camouflaging could play a role in this age difference of psychiatric disorders. While most studies did not find age differences in levels of camouflaging (Cook et al., 2021), one study found that older autistic adults showed less camouflaging compared with younger autistic adults (Perry et al., 2021). Therefore, we investigate whether age is an important factor in the relation between camouflaging and mental health.

Yet another important factor for camouflaging is someone’s cognitive capabilities. Autistic adults report that their intellect and executive functions enable them to learn camouflaging strategies (Livingston et al., 2019). However, studies that use quantitative measures for camouflaging indicate varying results about associations between camouflaging and cognitive abilities (Lai et al., 2016; Schuck et al., 2019). The role of cognitive abilities in the association between camouflaging and mental health problems has, to our knowledge not yet been investigated, but can help us better understand for whom camouflaging may be associated with mental health difficulties.

Aside from characteristics previously investigated in relationship to camouflaging and/or mental health, we also investigate the valence of someone’s emotional state. Although previous camouflaging research has not focused on the role of emotional state, research into coping strategies often takes emotional state into account. We can consider camouflaging as a specific coping strategy, since coping is defined as “constantly changing cognitive and behavioral efforts to manage specific external and/or internal demands that are appraised as taxing or exceeding the resources of the person” (Lazarus & Folkman, 1984). Mood can be an important moderator in the association between coping strategies and physical health (Adler & Matthews, 1994). According to the Broaden-and-Built Theory (Fredrickson, 2001), experiencing positive emotions broadens how someone thinks and behaves, and can increase creative problem solving and in the long run, makes someone more resilient and accepting. However, negative emotions narrow behavior and can result in a flight-or-fight response. Investigating emotional state could help us understand why camouflaging may be associated with mental health for some people but not for others.

In the present study, we aim to (1) replicate the finding that self-reported mental health difficulties and camouflaging are associated in autistic adults as described in Cook et al. (2021), (2) explore whether we find similar results when mental health difficulties are assessed with a clinical interview, and (3) investigate using a data-driven approach whether we can detect subgroups that show a different association between camouflaging and mental health, based on autism traits, biological sex, age, education, and emotional affect. With this, we aim to shed light on a promising mechanism behind mental health difficulties in autistic adults, which could eventually help in improving mental health care.

## Methods

### Participants

A total of 352 autistic adults aged 30–92 years participated in this study, of which a subgroup of autistic people (*N* = 161) was invited for an additional interview part of the study. Autistic adults were recruited through mental health institutions across the Netherlands, by means of advertisement via client organization websites and newsletters, and via social media (i.e. Twitter and LinkedIn). In addition, autistic adults aged ⩾30 who participated in previous studies were invited to participate in this study (Lever & Geurts, 2016). Inclusion criteria were (1) a self-reported clinical autism diagnosis, (2) no intellectual disability, and (3) understanding of the Dutch language. Additional inclusion criteria for the interviewed subgroup were (1) no history of neurological disorders (e.g. epilepsy, stroke, multiple sclerosis), schizophrenia, or having experienced more than one psychosis and (2) no current alcohol or drugs dependency. Participant characteristics are shown in Table 1. Please note that it is not standard to ask about race in the Netherlands. Only when it is crucial for a specific hypothesis, one is allowed to record race and/or ethnicity. However, participants are mostly White.

**Table 1.** Characteristics of all participants and the subgroup.

	Total (*N* = 352)		Subgroup (*N* = 161)	
	*M* (*SD*)	Min–max	*M* (*SD*)	Min–max
Biological sex (m/f/o)	183/167/2	–	104/56/1	–
Age	52.3 (12.5)	30-84	53.6 (14.0)	30–84
Education^a^	14/92/145/98	–	14/53/137/106	–
AQ	34.5 (7.5)	10–48	33.8 (7.8)	13–48
SCL-90-R	170.0 (51.4)	93–397	163.9 (50.9)	93–320
Positive affect	28.9 (7.2)	10–50	28.9 (7.5)	10–48
Negative affect	21.2 (8.2)	10–47	19.8 (7.5)	10–44
	Subgroup (*N* = 161)^b^			
	*N* (%) Current		*N* (%) Lifetime	
Any mental health condition^c^	66 (41.0)		130 (80.8)	
Any mood condition	17 (10.6)		104 (64.6)	
Any anxiety condition	60 (37.3)		101 (62.7)	
Other conditions	8 (6.0)		42 (26.1)	

*M*: mean, *SD*: standard deviation, min: minimum, max: maximum, m/f/o: male/female/other, AQ: Autism Quotient, SCL-90-R: Symptom Checklist 90 revised.

a For comparison of educational level, we merged the first four levels to prevent (almost) empty cells.

b This information is only available for the subgroup that participated in the face-to-face session.

c For all separate classifications, see Table S1.

### Materials

#### Regression variables

##### Camouflaging

The Dutch Camouflaging Autistic Traits Questionnaire (CAT-Q-NL; Hull et al., 2019; van der Putten et al., 2023) is a self-report questionnaire that measures different types of camouflaging (25 items, range: 25–175). Participants indicate on a 7-point Likert-type scale whether they *strongly disagree* (1) to *strongly agree* (7) with each statement. A higher score indicates more camouflaging.

##### Mental health difficulties

The Symptom Checklist-90 Revised (SCL-90-R; Arrindell & Ettema, 2005; Derogatis, 1977) is a self-report questionnaire to measure current psychopathological symptoms and psychological distress (90 items, range: 90–450). A higher score represents more mental health difficulties.

##### Mental health conditions

The Mini International Neuropsychiatric Interview Plus (MINI-plus; Sheehan et al., 1997; Van Vliet et al., 2000) is a structured diagnostic interview to measure current and lifetime mental health conditions according to the *Diagnostic and Statistical Manual of Mental Disorders* (4th ed.; DSM-IV). We did not include the classifications of alcohol abuse, substance abuse, psychotic disorders, and attention-deficit hyperactivity disorder (ADHD), as these were the exclusion criteria. Summing these classifications results in a “total current score” and “total lifetime score,” both ranging from 0 to 18.

#### Partitioning variables

##### Autism traits

The Autism Spectrum Quotient (AQ; Baron-Cohen et al., 2001; Hoekstra et al., 2008) is a self-report questionnaire that measures autism traits and consists of 50 items scored on a 4-point Likert-type scale. Items are rescored to a 0 or 1 following the AQ-algorithm, with a higher score representing more autism traits (50 items, range: 0–50).

##### Emotional affect

The Positive and Negative Affect Schedule (PANAS; Watson et al., 1988) is a self-report questionnaire to measure positive affect (e.g. strong, proud, and enthusiastic; 10 items, range: 10–50) and negative affect (e.g. upset, hostile and irritable; 10 items, range: 10–50). Higher scores on the PANAS represent more positive or negative affect.

##### Cognitive abilities

We included educational level as a proxy for cognitive abilities. Educational level was obtained by asking participants about their highest obtained educational degree. We used the Dutch Verhage scale to classify educational level ranging from 1 (<6 years of primary education) to 7 (university degree) (Verhage, 1964).

### Procedure

The present study is part of a large longitudinal study investigating “Autism & Aging” (Geurts et al., 2021) and approved by the ethical commission of the University of Amsterdam (2018-BC-9285). Participants gave written consent for participation in the study and completed two series of questionnaires. Hereafter, a subgroup was invited to face-to-face sessions during which, among others, the MINI-plus was administered. Participants were selected to participate in the face-to-face sessions so that there was an equal distribution of age and a sex ratio of around 2:1 in this subgroup due to the aims of the overarching study. Participants received compensation for travel expenses and a small monetary reward for participating in the study. Data used in this study are also used for other research questions (e.g. Radhoe et al., 2021; van der Putten et al., 2023).

### Community involvement

Four autistic and/or older adults were involved in the “Autism & Aging” study to advise, among others, on the recruitment of participants, study design, information letters, and interpretation of results. Researchers meet the group about four times a year over a period of 5 years and the group is compensated for their contributions. For this study, we discussed our results and how they experienced the relation between camouflaging and mental health in their own lives.

### Statistical analyses

Our analyses were preregistered at https://aspredicted.org/9B8_WJG. While we preregistered to compare autistic and non-autistic adults, we report only the findings of autistic adults. It was shown that comparing autistic and non-autistic adults using the CAT-Q-NL is not meaningful, because measurement invariance was violated (van der Putten et al., 2023). Therefore it is unclear what construct we would be measuring in non-autistic adults. Thus, we report only the findings for the autistic group, because the main goal of this study was to investigate the association between camouflaging and mental health for autistic adults. For transparency, we included the results of non-autistic adults in the Supplementary materials. In addition, in the preregistration the dependent and independent variables in the regression models were mistakenly switched. We executed the analyses as we originally intended and as would be logical for this research question, with camouflaging as independent variable and mental health difficulties as dependent variable.

Analyses were executed with Rstudio 3.6.2 (RStudio Team, 2020), using the “MobForest” package (Garge et al., 2013). First, we test the association between camouflaging and mental health using Pearson correlations between CAT-Q-NL score and SCL-90-R score and MINI-plus score (current and lifetime). Second, model-based recursive partitioning (MBRP) analyses were run. MBRP is a type of tree-structured regression analysis that can be used to identify whether regression models differ between subgroups by analyzing whether intercepts and regression coefficients differ for certain partitioning variables (Seibold et al., 2016; Zeileis et al., 2008). In this way, MBRP creates subgroups with similar relations between variables. Through MBRP, a fluctuation test for parameter instability is performed to assess whether splitting a subgroup improves the model. If there is significant parameter instability with respect to any of the partitioning variables, a split is made on the most instable variable. If no more significant instabilities can be found, the recursion stops and returns a tree where each subgroup has a different slope or intercept in the regression model. This procedure can be summarized in the following algorithm: (1) Fit the model once to all observations in the current (sub)group according to the specified regression model; (2) assess whether the parameter estimates are stable. If there is instability, select the variable associated with the highest parameter instability, but if there is no instability, stop; (3) compute the split point(s) that optimize the parameter; (4) split the group into subgroups and repeat the procedure. For an explanation of this procedure see Zeileis et al. (2008). *p*-values of 0.05 for testing significance of the partitioning variables were Bonferroni-adjusted to control for multiple comparisons and subgroups had to consist of minimally 20 individuals. We tested a linear model and included standardized SCL-90-R score as the dependent variable and CAT-Q-NL score as the independent variable. The included partitioning variables were autism traits (0–50), biological sex (male/female/other), age (in years), positive affect (10–50), negative affect (10–50), and educational level (1–7).

In addition, to explore whether we can find similar subgroups when mental health difficulties are measured using clinical interviews, we executed MBRP analyses as previously described, with MINI-plus score (current and lifetime) as a dependent variable. These analyses are executed in the subgroup (*N* = 161) for whom the MINI-plus is administered. Finally, to validate whether the partitioning variables in our regression trees are actually the most important predictors of the association between camouflaging and mental health difficulties, we executed Random Forest analyses. With random forest analyses, 300 MBRP analyses are executed on random subsamples of the data and with random subsets of partitioning variables.

## Results

### More camouflaging is associated with more mental health difficulties in autistic adults

Camouflaging and mental health difficulties (SCL-90-R) are positively correlated (*r* = 0.45, *p* < 0.001). This indicates that more camouflaging is associated with more self-reported mental health difficulties. Camouflaging was also positively associated with the number of mental health conditions currently (*r* = 0.30, *p* < 0.001) and lifetime (*r* = 0.40, *p* < 0.001), measured using a clinical interview (i.e. MINI-plus). All correlation coefficients between mental health measures, camouflaging, and the partitioning variables are shown in Table 2.

**Table 2.** Correlations between outcome and partitioning variables.

	1.	2.	3.	4.	5.	6.	7.	8.
1. CAT-Q-NL	–							
2. SCL-90-R	0.45**	–						
3. Biological sex^a^	0.12*	0.15*	–					
4. Age	–0.21**	–0.20**	–0.28**	–				
5. AQ	0.29**	0.29**	0.11*	0.04	–			
6. Negative affect	0.44**	0.80**	0.18	–0.22**	0.21**	–		
7. Positive affect	–0.17*	–0.20**	0.00	0.07	–0.26**	–0.15*	–	
8. MINI-current^b^	0.30**	0.51 **	0.07	–0.18*	0.30**	0.48**	–0.23**	–
9. MINI-lifetime^b^	0.40**	0.52**	0.21*	–0.22**	0.32**	0.48**	–0.22**	0.69**

CAT-Q-NL: Dutch Camouflaging Autistic Traits Questionnaire; SCL-90-R: Symptom Checklist-90 Revised; AQ: Autism Spectrum Quotient; MINI: Mini International Neuropsychiatric Interview Plus.

a Participants who indicated their biological sex as “other” (*N* = 2) were excluded from these analyses as this number was too small. Male = 1, female = 2.

b Only for the subgroup for whom the MINI was administered.

* *p* < 0.05. **p < 0.001.

### Negative affect is the most important variable for the association between camouflaging and self-reported mental health difficulties

**Figure 1.**
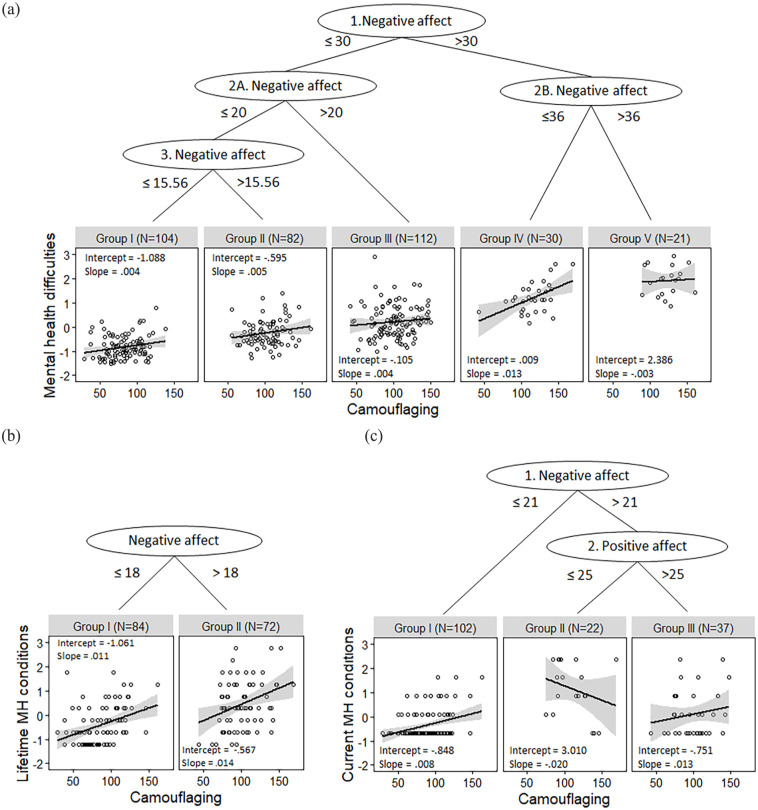
Visualization of subgroups based on model-based recursive partitioning analyses for the association between camouflaging (CAT-Q-NL) and (a) standardized self-reported mental health difficulties (SCL-90-R), (b) standardized lifetime mental health conditions (MINI-lifetime), and (c) standardized current mental health conditions (MINI-current).

Using MBRP analyses we investigated whether the intercepts and slopes differed in the association between camouflaging and self-reported mental health difficulties, based on autism traits, biological sex, age, educational level, and positive and negative affect. Test statistics of MBRP analyses are shown in Table 3 and the resulting regression trees are visualized in Figure 1. In total, we found five subgroups with different associations between camouflaging and mental health difficulties. These subgroups were solely characterized by different levels of negative affect and not by AQ, biological sex, age, educational level, or positive affect.

**Table 3.** Results of model-based recursive partitioning analyses for subgroups with N>40 (see note: 3) for the association between camouflaging and SCL-90-R, MINI-current, and MINI-lifetime.

Level^a^	Parameter instability statistics of partitioning variables						Split
	AQ	BioSex	Age	Edu	PA	NA	
SCL-90-R (Figure 1a)							
1	30.13***	6.79	9.31	3.80	9.59	142.74***	NA ⩽ 30
2A	11.48	8.44	11.67	2.79	10.51	73.06***	NA ⩽ 20
2B	10.07	4.03	4.38	1.90	7.57	15.92*	NA ⩽ 36
3	6.97	6.76	5 .70	5.20	9.31	28.32***	NA ⩽ 15.56
Group I	3.35	11.54	6.78	9.16	7.81	5.89	No
Group II	5.59	2.30	2.42	3.32	3.64	3.33	No
Group III	7.42	7.33	6.86	6.16	5.35	8.43	No
MINI-current (Figure 1b)							
1	18.12*	9.15	11.89	3.00	8.40	25.48***	NA ⩽ 21
2	9.54	3.60	5.39	2.29	15.69*	5.25	PA ⩽ 25
Group I	5.53	2.54	2.20	1.53	4.14	2.57	No
MINI-lifetime (Figure 1c)							
1	10.91	11.73	8.08	8.26	7.76	23.82**	NA ⩽ 18
Group I	5.13	3.53	9.00	11.17	5.21	5.20	No
Group II	7.99	3.39	6.50	3.91	5.24	4.72	No

SCL-90-R: Symptom Checklist-90 Revised; MINI: Mini International Neuropsychiatric Interview Plus; AQ: Autism Spectrum Quotient, BioSex: Biological Sex; PA: positive affect; NA: negative affect.

(1) This table corresponds to Figure 1 and shows for every partitioning variable, the statistics of the fluctuation tests and significance level. (2) The split for which most variance is explained is included in the regression tree and the criterion for this split is reported in the column “split.” (3) Subgroups should have a minimal size of 20. Therefore, in subgroups with *N* < 40, it is not tested whether a next split is possible and these are not included in the Table.

a The number indicates the level a split has been made on. The letter indicates the order of different splits within one level.

* *p* < 0.05. **p < 0.01. ***p < 0.001.

For some subgroups (I, II, and III) only the intercepts differed. This means that with a similar camouflaging score, there are more self-reported mental health difficulties, but the strength of the association did not differ. In these subgroups, camouflaging and mental health difficulties are related in a similar manner, that is, weakly. However, for Subgroups IV and V the slopes differed, which implies that with an increase in camouflaging, there is a different increase in mental health difficulties. We found that Subgroup IV has the highest slope and thus the strongest association. This subgroup has a relatively high score on negative affect (NA > 30, split 1), but not the highest negative affect (NA > 36, split 2B; group V). Finally, Subgroup V is characterized by the highest level of negative affect, has a high intercept but a low negative slope. Thus, in this subgroup people report high mental health difficulties but an increase in camouflaging is only weakly associated with a further decrease in mental health difficulties. In sum, the relationship between camouflaging and mental health especially differs based on negative affect and the relationship is only strong for Group IV.

Furthermore, Table 3 shows that biological sex, age, and educational level never came forward as significant split variables. However, AQ was a significant partitioning variable (Table 3, SCL-90-R, Level 1), but was not included as partitioning variable in the regression tree because a split based on negative affect explained more variance. To explore how autism traits affect the association between camouflaging and mental health, we repeated the MBRP analysis without negative affect in addition to our preregistered analyses. This resulted in two subgroups based on the level of autism traits, in which the subgroup with an AQ score higher than 41 had a stronger association compared with the subgroup with a lower AQ score (AQ ⩽ 41: intercept: −1.10, slope: 0.01, *N* = 287; AQ > 41: intercept: −1.70, slope: 0.03, *N* = 62). This indicates that there is a stronger association between camouflaging and mental health difficulties for people with more autism traits.

### Exploratory: the differences among subgroups

To gain better insight into the subgroups based on the SCL-90-R (Figure 1(a)), we tested group differences on partitioning and regression variables; see Table 4. Especially self-reported mental health difficulties differed across all groups, as well as negative affect. Also, in general camouflaging was higher in Subgroups IV and V compared with other subgroups. In addition, correlation coefficients and *R*^2^ for each subgroup separately indicate that in Subgroup IV camouflaging and mental health are the strongest associated and most variance is explained by the regression model in this subgroup.

**Table 4.** Characteristics of subgroups of autistic adults formed by MBRP analysis between camouflaging and self-reported mental health difficulties.

	Group I	Group II	Group III	Group IV	Group V	Test statistic	Post hoc difference
Biological sex (M/F/O)	62/42/0	52/29/2	47/65/0	9/21/0	10/11/0	χ^2^(4) = 18.33**	I vs. III, IV, II vs. III, IV
Age in years	56.1 (13.1, 30–81)	52.3 (11.9, 30–84)	49.8 (11.9, 30–81)	48.1 (11.2, 30–72)	49.5 (11.5, 30–70)	*F*(4,344) = 4.81***	I vs. III, IV
Education	40/38/26	26/35/21	30/46/37	6/14/10	5/12/4	χ^2^(4) = 13.70	
Positive affect	30.0 (7.6, 10–50)	28.3 (6.5, 17–46)	29.4 (7.2, 12–49)	27.2 (7.2, 15–42)	24.1 (5.21, 14–31)	*F*(4,344) = 3.80**	I vs. V, III vs. V
Negative affect	12.8 (1.7, 10–15.6)	17.8 (1.3, 15.6–20)	24.8 (2.8, 21–30)	33.3 (1.9, 31–36)	40.1 (2.60, 37–47)	*F*(4,344) = 1,184***	All groups differ
AQ	32.5 (8.6, 10–47)	34.5 (6.0, 17–46.9)	34.9 (7.0, 14–48)	37.3 (6.9, 21.5–48)	38.4 (6.9, 24–48)	*F*(4,344) = 4.78***	I vs. IV, V,
CAT-Q-NL	82.4 (22.5, 29–139)	102.4 (21.9, 55–162)	102.2 (25.2, 42–150)	116.4 (24.3, 43–169)	121.8 (18.9, 90–161)	*F*(4,344) = 23.53***	I vs. II, III, IV, V II vs. V, III vs. IV, V
SCL-90-R	127.2 (23.9, 93–211)	157.3 (27.6, 104–242)	182.4 (37.3, 118–337)	233.2 (32.4, 179–304)	277.6 (42.7, 214–397)	*F*(4,344) = 147.2***	All groups differ
Association CATQ/SCL							
Correlation	0.21*	0.20	0.15	0.51**	–0.07		
*R* squared	0.04	0.07	0.01	0.24	–0.05		

M/F/O: Male/Female/Other; AQ: Autism Spectrum Quotient; CAT-Q-NL: Dutch Camouflaging Autistic Traits Questionnaire; SCL-90-R: Symptom Checklist-90 Revised.

We reported mean (*M*) (standard deviation (*SD*); range) when appropriate.

* *p* < 0.05. **p < 0.01. ***p < 0.001.

### Similar variables are important when mental health conditions are measured with a clinical interview

To compare whether we find similar partitioning variables when we investigate the association between mental health conditions measured using the MINI-plus, we repeated the MBRP-analyses with current- and lifetime-MINI as dependent variables. The resulting subgroups are shown in Figure 1(b) and (c). While the criteria for splits differ, partitioning variables in the regression trees are similar to the ones found for self-reported mental health difficulties.

### Negative affect is consistently the most important partitioning variable

Random Forest analyses (see method: statistical analyses) show that negative affect was consistently the most important partitioning variable, followed by AQ-score and positive affect. Biological sex, age, and educational level were not important partitioning variables. These results were similar to MINI-current and lifetime as outcome variables. This indicates that we can reliably conclude that biological sex, age, and educational level are not important factors to consider in the association between camouflaging and mental health difficulties, but negative affect, autism traits, and positive affect are.

## Discussion

In the present study, we used a data-driven approach to gain a deeper understanding in the association between camouflaging and mental health by investigating whether we could detect subgroups of autistic adults that showed differences in this association. Our findings shed a more nuanced light on the dominant hypothesis that camouflaging is associated with mental health difficulties in autistic adults (Cook et al., 2021). Plainly stating that camouflaging and mental health are negatively associated for all autistic people is therefore an oversimplification. This is in line with the widely acknowledged heterogeneity in autism (Agelink van Rentergem et al., 2021; Happé & Frith, 2020).

There are three main conclusions that we can draw from this study. First, we observed that there is indeed a moderate association between camouflaging and self-reported mental health difficulties when investigating the total group of autistic adults. Second, this relationship is also found between camouflaging and mental health conditions measured using a clinical interview. We showed that people who report more camouflaging behavior also meet the criteria of more mental health conditions currently (at the time of the interview), but also during their life. However, the third finding nuances these first two conclusions, as the strength of the relationship between camouflaging and mental health difficulties differed across subgroups and was mainly strong for one subgroup. The subgroups with a stronger association between camouflaging and mental health difficulties were characterized by higher levels of negative affect or autism traits. We did not find differences in the association between camouflaging and mental health difficulties based on biological sex, age, or educational level. While the level of camouflaging has been found to differ based on someone’s biological sex, age, or cognitive capacities (Cook et al., 2021), these characteristics by themselves do not seem to explain whether camouflaging is related to mental health difficulties. Therefore, we conclude that someone’s (negative) emotional state or autism traits are most important for the relationship between camouflaging and mental health.

For most autistic adults, we found that the relationship between camouflaging and self-reported mental health difficulties is small (Subgroup I), small but nonsignificant (Subgroups II and III), or non-existent (Subgroup V). We only found a strong relationship in Subgroup IV, which consisted of 10% of all participants. For this subgroup, camouflaging explained most variance in mental health. This implies that camouflaging is very important for mental health in this specific subgroup, but less important for other subgroups. It is possible that the association in the total group is inflated by the strong association in this small subgroup, while there seems to be only a weak association for most people as reflected in the mild relationships in the other subgroups. Interestingly, in Subgroup V, characterized by the highest negative affect and mental health difficulties, we found a weak negative association. This implies that camouflaging is not always important when people have many mental health difficulties. For this subgroup, there may be other causes for their mental health difficulties that are more important to focus on than camouflaging.

The factor that complicates interpretation of the subgroups is that all splits were made based on negative affect and that negative affect and mental health difficulties are highly correlated. Although constructs differ conceptually, a high association was also found in the PANAS validation study (Watson et al., 1988). Also, in the *Diagnostic and Statistical Manual of Mental Disorders* (5th ed., text revised; DSM-5-TR; American Psychiatric Association, 2022), experiencing long periods of negative affect is regarded as a transdiagnostic symptom of depression. Therefore, it is difficult to disentangle whether Subgroups I, II, and III meaningfully differ in the association between camouflaging and mental health, since only their intercepts differ. However, we do know that both Subgroups IV and V differ from other subgroups in the association between camouflaging and mental health. That is, we found that slopes varied compared with other subgroups and thus the strength of the association differs between these subgroups. Furthermore, while we talk about subgroups here, one should be careful in referring to the observed partitions as valid and reliable, as it was not the goal of the current study to test the factual properties of these subgroups (see for a discussion about subgroups validity: Agelink van Rentergem et al., 2021).

Finally, one might think that negative affect overshadowed the effect of biological sex, age, or educational level, but this is not the case. Through MBRP analyses, we investigated for every variable separately whether the association between camouflaging and mental health differed based on this variable. The variable that explained most variance was included in the regression tree (Zeileis et al., 2008). Since biological sex, age, and educational level never came forward as a significant split, we conclude these are not relevant factors. However, autism traits were a significant predictor and this split may have been overshadowed by negative affect. When we excluded negative affect from the analyses, we indeed found a stronger association for the subgroup with higher autism traits compared with lower autistic traits. Therefore, we conclude that negative affect and autism traits play an important role in the association between camouflaging and self-reported mental health difficulties.

The main strength of this study is that we were able to investigate the association between camouflaging and mental health in a very broad manner because of our sample, measurements and statistical analysis. Our sample consisted of a large and sufficiently heterogeneous group of autistic adults in a wide age range with a large number of male and female participants. In addition, we used different well-validated measures to get a clear overview of mental health difficulties and conditions that autistic adults experience. Finally, by using a data-driven statistical analysis, we could uncover the most important predictors for the relationship between camouflaging and mental health instead of specifying beforehand which predictor variable would be most important. However, since the analyses were data-driven, it is important to replicate our findings in independent samples.

The current study is not without caveats. The main limitation is that we could not execute our full preregistered analysis plan by investigating this relationship in non-autistic adults, since it is difficult to interpret what CAT-Q-NL-scores mean for non-autistic adults. Also, we operationalized cognitive capacities by educational level and therefore have to limit our conclusions to statements about educational level, since educational level may have been influenced by more than cognitive capacities alone. In addition, participants all had a middle to high educational level. Whether educational level is relevant for camouflaging and mental health deserves future studies with more variation in educational level. Since we included only autistic people without an intellectual disability, these findings do not generalize to autistic people with an intellectual disability. Whether camouflaging occurs in this group or is associated with mental health difficulties, could be further investigated. Also, in the present study, we focused on biological sex, not on gender. A previous study showed that gender diverse autistic people reported more compensation strategies than cisgender autistic people (McQuaid et al., 2022). In that study, it was hypothesized that gender diverse people may be influenced more by gender stereotypes and therefore might be more inclined to show camouflaging behavior. Examining whether the association between camouflaging and mental health differs for gender diverse autistic people could be an important next step. Furthermore, longitudinal studies are necessary to draw conclusions about causality and contextual information could help inform for whom and when camouflaging is related to mental health difficulties. Finally, specific measures of potential positive consequences could shed further light on for whom and when camouflaging can be helpful.

### Clinical implications

Our findings can already be informative for mental health care. That is, an autistic person who experiences a substantial level of negative emotions, for example, being irritable or distressed, could be in Subgroup IV, for which there is a strong relationship between camouflaging and mental health. If this person also struggles with mental health difficulties, it would be useful to explore if and why camouflaging and mental health difficulties are related and whether it is possible to focus on this association. However, when someone expresses many strong negative emotions and many mental health difficulties, this person may belong to Subgroup V, for which mental health and camouflaging does not seem to be associated. Therefore, for this subgroup, camouflaging would not be the first factor to focus on when providing mental health care. Owing to the cross-sectional nature of this study, we can only speculate about the directions of the relationships found in the present study. However, in clinical practice we advise to try to disentangle the relationship between camouflaging behavior and mental health difficulties, especially for people who report substantial levels of negative affect. This may provide insights into if and why camouflaging is associated with mental health difficulties for a specific individual.

## Conclusion

In sum, based on the present study, we conclude that camouflaging and mental health are associated for some autistic adults, but not for everyone. Whether camouflaging and mental health are associated varies based on someone’s emotional state and autism traits, but it does not depend on biological sex, age, or educational level. Therefore, camouflaging can be important to consider in clinical practice, especially in combination with negative affect, but one needs to be careful with group-based conclusions regarding the (negative) impact of camouflaging.

## Supplemental Material

sj-docx-1-aut-10.1177_13623613231185402 – Supplemental material for The relationship between camouflaging and mental health: Are there differences among subgroups in autistic adults? Supplemental material, sj-docx-1-aut-10.1177_13623613231185402 for The relationship between camouflaging and mental health: Are there differences among subgroups in autistic adults? by Wikke J van der Putten, Audrey JJ Mol, Tulsi A Radhoe, Carolien Torenvliet, Joost A Agelink van Rentergem, Annabeth P Groenman and Hilde M Geurts in Autism

## Notes

1. In this article, the term autism is used instead of “autism spectrum disorder” and identity-first language is used instead of person-first language (Bury et al., 2020; Kenny et al., 2016).

2. In this article we use the term camouflaging, but different terms are currently used, such as masking, compensating, passing as non-autistic, and adaptive morphing (Lawson, 2020; Libsack et al., 2021).
