# Supplementary material for: The relationship between camouflaging and mental health: Are there differences among subgroups in autistic adults?
Source: Autism. 2023 Jul 27;28(4):908–19. doi: 10.1177/13623613231185402 (PMC10981194; doi:10.1177/13623613231185402)
Supplement: sj-docx-1-aut-10.1177_13623613231185402 – Supplemental material for The relationship between camouflaging and mental health: Are there differences among subgroups in autistic adults? [file sj-docx-1-aut-10.1177_13623613231185402.docx]

Supplementary materials for:

**The relationship between camouflaging and mental health: Are there differences among subgroups in autistic adults?**

**Table S1.**
*Lifetime and current frequency of mental health conditions in autistic adults.*

|  | Autism subgroup (N=161) ^a^ | |
| --- | --- | --- |
|  | N (%) Lifetime | N (%) Current |
| Any mental health condition | 130 (80.8%) | 66 (41.0%) |
| *Mood disorders* |  |  |
| Major depressive episode | 101 (62.7%) | 14 (8.7%) |
| Dysthymia | 29 (18.0%) | 4 (2.5%) |
| *Anxiety disorders* |  |  |
| Panic disorder | 18 (11.2%) | 3 (1.9%) |
| Agoraphobia | 39 (24.2%) | 31 (19.3%) |
| Social phobia | 55 (34.2%) | 28 (17.4%) |
| Specific phobia | 22 (13.7%) | 12 (7.5%) |
| OCD | 30 (18.6%) | 17 (10.6%) |
| PTSD | 16 (10.0%) | 3 (1.9%) |
| GAD | 32 (19.9%) | 18 (11.2%) |
| *Other conditions* | 42 (26.1%) | 8 (6.0%) |

Note. OCD: obsessive compulsive disorder, PTSD: posttraumatic stress disorder, GAD: generalized anxiety disorders, other conditions: either (hypo)manic episode, anorexia nervosa, bulimia nervosa, somatization disorder, hypochondriasis, body dysmorphic disorder, pain disorder, adjustment disorders and mixed anxiety-depressive disorder.
^a^ Information is available for the subgroup that participated in the face-to-face session (N = 161) and not for the whole group.

**The relationship between camouflaging and mental health: Are there differences among subgroups in non-autistic adults?**

Please note that we do not interpret the findings in the non-autistic group as our earlier research showed that it is not evident what the CAT-Q-NL precisely measures in a non-autistic group. The current results are solely reported to be transparent as we preregistered our analyses before we knew the problems with the CAT-Q-NL in the non-autistic group.

**Participants**

A total of 312 non-autistic adults aged 30 to 92 years participated in this study. Non-autistic adults were recruited through social media and personal networks of researchers and participants. In addition, non-autistic adults aged 30 or higher that participated in previous studies were also invited to participate in this study as was described in. Characteristics of participants are shown in Table S2. Inclusion criteria for all non-autistic participants were: 1) no intellectual disability, 2) sufficient understanding of Dutch language, 3) no present or past autism or Attention Deficit Hyperactivity Disorder (ADHD) diagnosis, 4) no direct family members with an autism or ADHD-diagnosis and 5) AQ ≤ 32 and DSM-IV criteria during childhood and adulthood for inattention and hyperactivity/impulsivity < 6 based on the ADHD-Rating Scale (ADHD-SR; Kooij et al., 2005)). Additional inclusion criteria for the subsample that was invited for a subsequent face-to-face session were 1) no history of neurological disorders (e.g., epilepsy, stroke, multiple sclerosis), schizophrenia or having experienced more than one psychosis and 2) no self-reported current alcohol or drugs dependency.

**Table S2.**
*Characteristics of all non-autistic participants.*

|  | COMP (N=312) | | COMP subgroup (N=137) | |
| --- | --- | --- | --- | --- |
|  | M (sd) | Min-max | M (sd) | Min-max |
| Sex (m/f/o) | 169/143/0 | - | 86/51/0 | - |
| Age | 56.7 (13.8) | 30-92 | 56.3 (14.3) | 30-85 |
| Education ^a^ | 67/137/106 | - | 21/65/51 | - |
| AQ total | 13.8 (5.8) | 3-31 | 14.1 (6.2) | 3-30 |
| SCL-90-R total | 113.5 (21.2) | 90-213 | 116.1 (23.1) | 91-213 |
| Positive affect | 35.7 (4.9) | 16-48 | 35.5 (5.2) | 16-48 |
| Negative affect | 14.0 (4.1) | 10-37 | 14.6 (4.6) | 10-37 |
|  | COMP (N=137) ^b^ | | | |
|  | N (%) Lifetime | | N (%) Current | |
| Any mental health condition | 53 (38.7%) | | 9 (6.7%) | |
| *Mood conditions* | | | | |
| Major depressive episode | 36 (26.3%) | | 0 (0%) | |
| Dysthymia | 5 (3.7%) | | 0 (0%) | |
| *Anxiety conditions* | | | | |
| Panic disorder | 4 (2.9%) | | 0 (0%) | |
| Agoraphobia | 10 (7.3%) | | 1 (.7%) | |
| Social phobia | 9 (6.6%) | | 1 (.7%) | |
| Specific phobia | 5 (3.7%) | | 1 (.7%) | |
| OCD | 3 (2.2%) | | 2 (1.5%) | |
| PTSD | 3 (2.2%) | | 1 (.7%) | |
| GAD | 5 (3.7%) | | 2 (1.5%) | |
| *Other conditions* | 10 (7.3%) | | 1 (.7%) | |

Note. Note. COMP: comparison, M: mean, SD: standard deviation, min: minimum, max: maximum, m/f/o: male/female/other, AQ: Autism Quotient, SCL-90-R: Symptom Checklist 90 revised, OCD: obsessive compulsive disorder, PTSD: posttraumatic stress disorder, GAD: generalized anxiety disorders, other conditions: either (hypo)manic episode, anorexia nervosa, bulimia nervosa, somatization disorder, hypochondriasis, body dysmorphic disorder, pain disorder, adjustment disorders and mixed anxiety-depressive disorder.
^a^ For comparison of educational level we merged the first four levels to prevent (almost) empty cells.
^b^ Information is available for the subgroup that participated in the face-to-face session (N = 137) and not for the whole group.

**Results**All correlation coefficients between mental health measures, camouflaging, and the partitioning variables, are shown in Table S3. Test statistics of MBRP-analyses are shown in Table S4 and the resulting regression trees are visualized in Figure S1.

|  | 1. | 2. | 3. | 4. | 5. | 6. | 7. | 8. |
| --- | --- | --- | --- | --- | --- | --- | --- | --- |
| 1. CAT-Q-NL | - |  |  |  |  |  |  |  |
| 2. SCL-90-R | .30** | - |  |  |  |  |  |  |
| 3. Biological sex ^a^ | -.12* | .02 | - |  |  |  |  |  |
| 4. Age | -.04 | .02 | -.03 | - |  |  |  |  |
| 5. AQ | .25** | .29** | -.20** | .16* | - |  |  |  |
| 6. Negative affect | .31** | .73** | .01 | -.12* | .15* | - |  |  |
| 7. Positive affect | -.21** | -.37** | .10 | -.06 | -.25** | -.16* | - |  |
| 8. MINI current ^b^ | .08 | .20* | .00 | -.11 | .06 | .15 | -.11 | - |
| 9. MINI lifetime ^b^ | .32** | .38** | -.10 | -.03 | .07 | .31** | -.06 | .26 |

**Table S3.**
*Correlations between all outcome and partitioning variables for non- autistic adults.*

*Note.* AQ: Autism Spectrum Quotient, CAT-Q-NL: Dutch Camouflaging Autistic Traits Questionnaire, MINI: Mini International Neuropsychiatric Interview Plus, SCL-90-R: Symptom Checklist-90 Revised, * = *p* <.05, ** = *p* < .001. ^a^ Male was coded as 1, female as 2. ^b^ The correlations with MINI scores are only for the subgroup for whom the MINI was administered.

**Table S4.**
*Results of Model-Based Recursive Partitioning analyses for subgroups with N > 40 (see: note: C) for the association between camouflaging and SCL-90-R, MINI-current, and MINI-lifetime in non-autistic adults.*

| Level ^a^ | Parameter instability statistics of partitioning variables | | | | | | Split |
| --- | --- | --- | --- | --- | --- | --- | --- |
|  | AQ | Sex | Age | Edu | PA | NA |  |
| SCL-90-R (Figure S1) | | | | | | |  |
| 1 | 21.92 ** | 5.50 | 3.33 | 9.71 | 30.46*** | 110.71*** | NA ≤ 16 |
| 2A | 32.77 *** | 6.28 | 6.95 | 5.51 | 29.14*** | 51.72 *** | NA ≤ 12 |
| 2B | 4.23 | 2.09 | 2.91 | 4.65 | 9.52 | 17.53* | NA ≤ 21 |
| 3A | 4.71 | 7.02 | 20.37 ** | 1.98 | 20.99** | 19.69 ** | PA ≤ 34 |
| 3B | 18.51 * | 1.35 | 5.41 | 6.72 | 13.73 | 8.10 | AQ ≤ 18 |
| Group II | 2.48 | 4.00 | 9.28 | 2.63 | 8.19 | 10.13 | No |
| Group III | 4.02 | 2.01 | 3.03 | 10.05 | 3.82 | 7.11 | No |
| Group V | 5.65 | 2.26 | 3.03 | 1.66 | 8.63 | 3.56 | No |
| MINI current | | | | | | |  |
| Group I | 3.24 | 4.81 | 7.25 | 2.08 | 3.88 | 10.45 | No |
| MINI lifetime | | | | | | |  |
| Group I | 5.13 | 3.53 | 9.00 | 11.17 | 5.21 | 5.20 | No |

*Note.* A) This Table corresponds to Figure 1 and shows for every partitioning variable the statistics of the fluctuation tests and significance level. B) The split for which most variance is explained is included in the regression tree and the criterium for this split is reported in the column “split”. C) Subgroups should have a minimal size of 20. Therefore in subgroups with N<40, it is not tested whether a next split is possible and these are not included in the Table.
AQ: Autism Spectrum Quotient, MINI: Mini International Neuropsychiatric Interview Plus, NA: Negative affect, PA: Positive affect, SCL-90-R: Symptom Checklist-90 Revised. *: *p* <.05, **: *p* <.01, ***: *p* <.001. ^a^ The number indicates the level a split has been made on. The letter indicates the order of different splits within one level.

**Figure S1.**
*Visualization of subgroups based on Model-Based Recursive Partitioning analyses for the association between camouflaging (CAT-Q-NL) and standardized self-reported mental health difficulties (SCL-90-R) in non-autistic adults.*

**
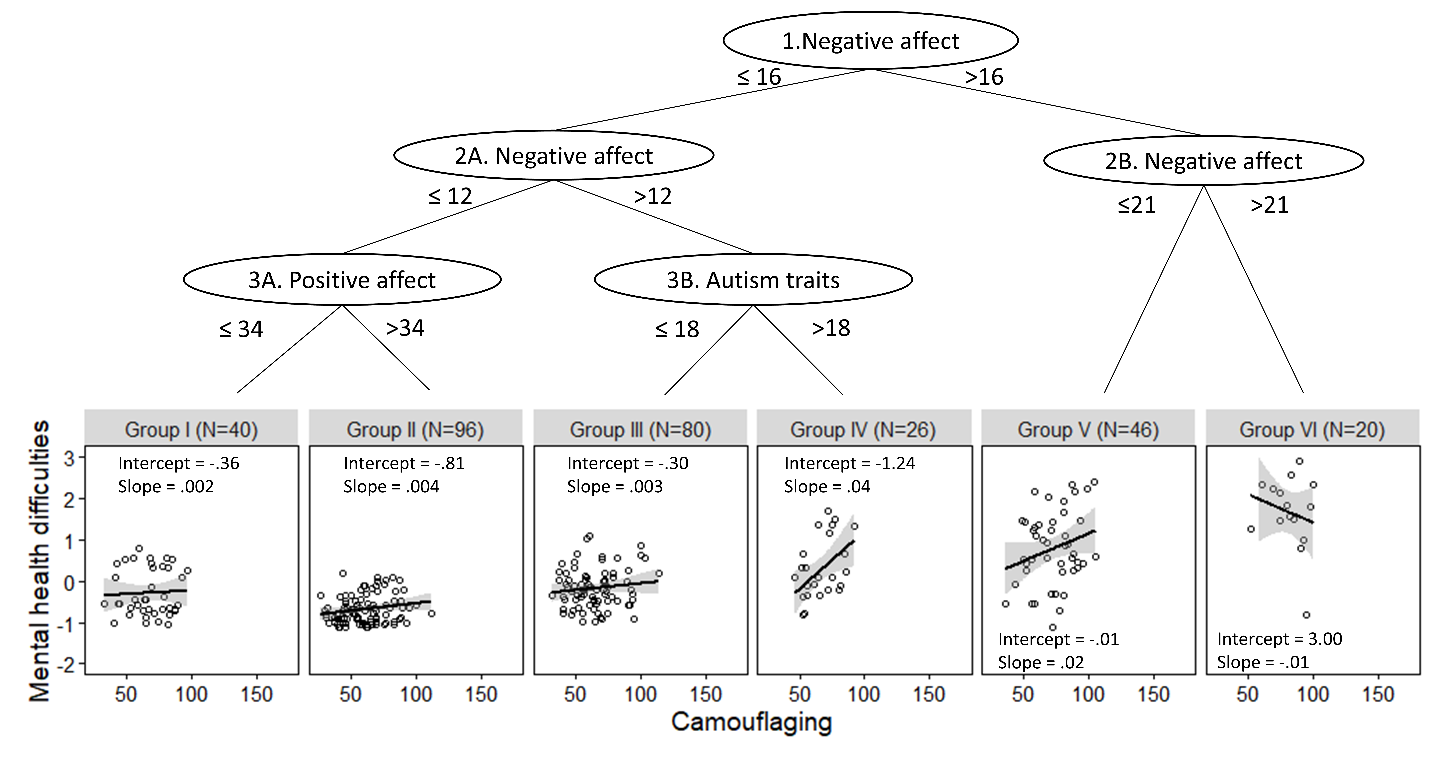
**
